# Long-Term Effects of Very Low Dose Particle Radiation on Gene Expression in the Heart: Degenerative Disease Risks

**DOI:** 10.3390/cells10020387

**Published:** 2021-02-13

**Authors:** Venkata Naga Srikanth Garikipati, Arsen Arakelyan, Eleanor A. Blakely, Polly Y. Chang, May M. Truongcao, Maria Cimini, Vandana Malaredy, Anamika Bajpai, Sankar Addya, Malik Bisserier, Agnieszka Brojakowska, Abrisham Eskandari, Mary K. Khlgatian, Lahouaria Hadri, Kenneth M. Fish, Raj Kishore, David. A. Goukassian

**Affiliations:** 1 Department of Emergency Medicine, Dorothy M Davis Heart and Lung Research Institute, Wexner Medical School, The Ohio State University, Columbus, OH 43210, USA; venkata.garikipati@osumc.edu; 2 Bioinformatics Group, The Institute of Molecular Biology, The National Academy of Sciences of the Republic of Armenia, Yerevan 0014, Armenia; arakelyanaa@gmail.com; 3 PathVerse, Yerevan 0014, Armenia; 4 Lawrence Berkeley National Laboratory, Berkeley, CA 94720, USA; eablakely@jbl.gov; 5 SRI International, Menlo Park, CA 94025, USA; polly.chang@sri.com; 6 Center for Translational Medicine, Lewis Katz School of Medicine, Temple University, Philadelphia, PA 19140, USA; maycao@temple.edu (M.M.T.); maria.cimini@temple.edu (M.C.); tuj39930@temple.edu (V.M.); nmkbjp18@gmail.com (A.B.); raj.kishore@temple.edu (R.K.); 7 Kimmel Cancer Center, Sidney Kimmel Medical College, Thomas Jefferson University, Philadelphia, PA 19107, USA; sankar.addya@jefferson.edu; 8 Cardiovascular Research Center, Icahn School of Medicine at Mount Sinai, New York, NY 10029, USA; malik.bisserier@mssm.edu (M.B.); agnieszka.brojakowska@mssm.edu (A.B.); abrisham.eskandari@icahn.mssm.edu (A.E.); mkhlgatian@gmail.com (M.K.K.); lahouaria.hadri@mssm.edu (L.H.); kenneth.fish@mssm.edu (K.M.F.)

**Keywords:** heart, space radiation, gene expression, gamma radiation, NASA, female mice

## Abstract

Compared to low doses of gamma irradiation (γ-IR), high-charge-and-energy (HZE) particle IR may have different biological response thresholds in cardiac tissue at lower doses, and these effects may be IR type and dose dependent. Three- to four-month-old female CB6F1/Hsd mice were exposed once to one of four different doses of the following types of radiation: γ-IR ^137^Cs (40-160 cGy, 0.662 MeV), ^14^Si-IR (4-32 cGy, 260 MeV/n), or ^22^Ti-IR (3-26 cGy, 1 GeV/n). At 16 months post-exposure, animals were sacrificed and hearts were harvested and archived as part of the NASA Space Radiation Tissue Sharing Forum. These heart tissue samples were used in our study for RNA isolation and microarray hybridization. Functional annotation of twofold up/down differentially expressed genes (DEGs) and bioinformatics analyses revealed the following: (i) there were no clear lower IR thresholds for HZE- or γ-IR; (ii) there were 12 common DEGs across all 3 IR types; (iii) these 12 overlapping genes predicted various degrees of cardiovascular, pulmonary, and metabolic diseases, cancer, and aging; and (iv) these 12 genes revealed an exclusive non-linear DEG pattern in ^14^Si- and ^22^Ti-IR-exposed hearts, whereas two-thirds of γ-IR-exposed hearts revealed a linear pattern of DEGs. Thus, our study may provide experimental evidence of excess relative risk (ERR) quantification of low/very low doses of full-body space-type IR-associated degenerative disease development.

## 1. Introduction

During future exploration-type space missions, astronauts could be exposed to doses of space-type radiation (IR) (~0.4–0.5 Gy) from galactic cosmic rays (GCR), especially during Mars missions, where astronauts would not have access to comprehensive health care services for at least 2–3 years [1,2]. Emerging evidence suggests that during deep-space missions, each cell in an astronaut’s body could be traversed by a proton every 3–4 days, helium nuclei every few weeks, and high-charge-and-energy (HZE) nuclei (e.g., C, O, Si, Ti, Fe) every few months [3,4]. Since most experienced astronauts are middle-aged (average age is 46 years, range is 33 to 58 years of age), they are at risk for developing severe, adverse cardiovascular (CV) and other degenerative diseases both during long space missions as well as later in life [5]. Others and our own NASA-funded published data provided experimental evidence of the effects of low-dose proton and HZE-IR on the short- and long-term alteration in cardiac function, cardiomyocyte morphology, and the associated underlying molecular mechanisms [6,7,8,9,10]. Here, we provide for the first time the transcriptome analysis of mouse hearts exposed to low and very low doses of gamma- (^137^Cs), silicon- (^14^Si), and titanium- (^22^Ti) ion irradiation (IR). Animal numbers per each irradiated group were selected by power analysis to assure statistical significance after low dose radiation exposures. Our results show that 16 months after a single low- or very low dose IR exposure, the gene expression in the heart tissue is significantly differentially regulated compared to the sham-treated, non-irradiated controls, suggesting there are long-term effects on dysregulation of varying molecular pathways that are associated with various degrees of CV, pulmonary, and metabolic diseases, as well as biological processes, including abnormal circadian rhythms, cancer, Hutchinson–Gilford progeria syndrome, etc. Thus, our study may provide additional experimental evidence of the level of gene expression for estimation of excess relative risk (ERR) for the development of CV and other diseases due to exposure to low or very low doses of whole-body space-type IR.

## 2. Materials and Methods

### 2.1. Animal Procedures

We used NASA Space Radiation Tissue Sharing Forum–archived samples of female CB6F1/Hsd mice from experiments conducted and published by Drs. Polly Chang and Eleanor Blakely for evaluation of IR-induced carcinogenesis in the mouse Harderian gland [11]. Detailed experimental protocols were previously published by Chang et al. [11]. Briefly, female CB6F1/Hsd mice were obtained from Harlan Laboratories Inc. (Indianapolis, IN, USA). For each study, 100–120-day-old female mice were shipped directly from the vendor to Brookhaven Laboratory Animal Facility (BLAF; Upton, New York, NY, USA) approximately 1 week before irradiation. Mice were randomized by weight immediately prior to whole-body particle IR exposure. After exposure, mice were shipped to the animal facility at the Lawrence Berkeley National Laboratory (LBNL) and maintained for 16 months. The 16-month period was chosen as it coincides with the time frame for Harderian gland tumor development. All procedures were compliant with the standards of the Guide for the Care and Use of Laboratory Animals of the National Institutes of Health and approved by the Animal Care and Use Committees at Lawrence Berkeley National Laboratory (Berkeley, CA, USA), Brookhaven National Laboratory (BNL) (Upton, NY, USA), and SRI International (Menlo Park, CA, USA, which included established diurnal room lights on/off cycles).

### 2.2. Irradiation Procedures

Detailed experimental protocols were previously published by Chang et al. [11]. Briefly, female CB6F1/Hsd mice (*n* = 5/group) were irradiated with low and very low doses of HZE-IR at the NASA Space Radiation Laboratory (NSRL) at the BNL. Dosimetry studies, including depth–dose and dose–uniformity measurements, were conducted by beamline physicists. Unanesthetized animals were briefly held in plastic boxes (40 × 40 × 73 mm^3^) containing numerous holes to provide abundant airflow. Animals were irradiated for <1 min with a single, full-body HZE-IR dose (≤0.5 Gy) of 260 MeV/n silicon (^14^Si, linear energy transfer [LET] ≈ 70 keV/µm) and 1000 MeV/n titanium (^22^Ti, LET ≈ 100 keV/µm). As another control group, a separate cohort of animals was irradiated with ^137^Cs γ-ray that served as a low-LET reference radiation. Table 1 depicts beam energies, doses, and LET used in these studies. Control sham-treated animals were placed in clear plastic holders for equivalent exposure times but were not irradiated. Similar to irradiated mice, control mice were also maintained unanesthetized.

### 2.3. Tissue Collection and Sample Processing

Animals were sacrificed 16 months after a single dose of full-body IR with the corresponding ions and doses (Table 1). Gross observations of all tissues were conducted during necropsy and recorded. Hearts from control non-tumor-bearing mice were snap-frozen in liquid nitrogen by members of Drs. Chang and Blakely’s research team [11] and archived as part of NASA’s Space Radiation Tissue Sharing Forum. These archived heart tissue samples were utilized for our current gene expression study.

### 2.4. RNA Extraction

Total RNA was isolated from snap-frozen heart tissues using the RNeasy Mini Kit (Qiagen, Germantown, MD, USA) according to the manufacturer’s protocol. Briefly, heart tissues were lysed and homogenized in the presence of a highly denaturing guanidine-thiocyanate-containing buffer, which immediately inactivates RNAses to ensure purification of intact RNA. Ethanol (70%) was added to provide appropriate binding conditions. The homogenates were then applied to a RNeasy Mini spin column, where the total RNA binds to the membrane and contaminants are efficiently washed away. High-quality RNA was eluted using molecular-grade water (RNAse/DNAse free). RNA concentrations were assessed using a Nanodrop ND-1000 spectrophotometer (NanoDrop Technologies, Wilmington, DE, USA). Quality assessment was performed using Agilent 2200 TapeStation (Agilent Technologies, Palo Alto, CA, USA) prior to qRT-PCR and microarray analysis.

### 2.5. Microarray Analysis

Linear messenger RNA (mRNA) amplification was achieved using the Affymetrix WT-Plus kit (Affymetrix, Santa Clara, CA, USA) according to the manufacturer’s guidelines. Briefly, 100 ng of RNA from each sample was used to generate amplified and biotinylated complimentary DNA (cDNA) according to the guidelines of the Affymetrix WT-Plus kit. Target denaturation was performed at 99 °C for 5 min. cDNA was then hybridized to the Clariom S Mouse Array (Affymetrix, Santa Clara, CA, USA) for 16 h at 45 °C while being rotated at 60 rpm. Arrays were then washed and stained using GeneChip Fluidics Station 450 (Affymetrix, CA, USA) and subsequently scanned with Affymetrix Gene Chip Scanner 3000 using Command Console software. Signal space transformation (SST) normalization of raw data was performed using Expression Console software v 1.41. (provided by Affymetrix). GeneSpring 14.9 software (Agilent, Santa Clara, CA, USA) was used to identify differentially expressed transcripts between experimental and control groups. Of note, samples were processed for microarrays in two batches: first, 3 samples for each condition (*n* = 36) and then 2 samples for each treatment condition (*n* = 24) for a total of 60 samples representing 5 animals per each ion/dose.

### 2.6. Batch Examination and Correction

Principal components analysis (PCA) was used to identify batch effects by examining patterns in plots of the first two principal components. Three methods for batch effect adjustment were used: (1) Combat implemented in the sva R package [12], (2) remove BatchEffect implemented in the limma R package [13], and (3) harman implemented in the Harman R package [14]. Changes in batch effect contribution after adjustment were assessed using the pvca R package [15].

### 2.7. Microarray Data Preprocessing

Background correction, conversion of signal intensity to log2-transformed expression values, and quantile normalization were performed on microarray raw CEL files using the Robust MultiArray Average (RMA) algorithm implemented in the limma R package [13]. Microarray probe IDs were converted to Entrez IDs using annotate and clariomsmousetranscriptcluster.db R packages.

### 2.8. Analysis of Differential Gene Expression

Differentially expressed genes (DEGs) between control and each irradiated group were identified using limma package [13]. Relaxed adjusted *p*-values of <0.25 were selected as a cutoff for up- and downregulated genes, which is reasonable for finding an adequate amount of DEGs for further validation and functional gene set analysis. Functional annotation of DEGs was performed with the enrichR R package, which contains 35 human and rodent gene set libraries (Supplementary Table S1) [16]. The Enrichr gene set enrichment method is based on a combined score calculated from a proportional test (Fisher’s exact test) and the *z*-score of deviation from the expected rank for each term in each gene set library (see original publications for details [16]). Adjusted *p*-values of < 0.05 were selected as a cutoff for over-represented gene sets. The lists of all differentially expressed genes are provided in Supplementary Table S2.

### 2.9. Analysis of Dose-Dependent Changes in Gene Expression

To evaluate the patterns of gene expression changes in response to increasing doses of irradiation, we performed linear and non-linear regression analysis using functions from stats and splines R packages, respectively. For each gene, both linear and non-linear regression models were fitted. The fit was considered significant if the model-adjusted *p*-value was less than 0.25, and the fit type was assigned based on the highest *R*-squared value between two models for each gene.

### 2.10. Portrayal of Transcriptome Landscapes

Transcriptome analysis was performed using the self-organizing map (SOM) machine learning approach implemented in the oposSOM R package [17]. The SOM approach represents dimension reduction that translates 20,426 gene expression profiles in 72 samples into 1600 metagenes, each representing a cluster of genes with similar profiles of expression across samples. The metagene expression values of each sample are visualized (expression portrayal) by arranging them into a two-dimensional 40 × 40 grid and using maroon to blue colors corresponding to maximum to minimum expression values in each of the portraits [18]. The oposSOM R package also offers a variety of tools for downstream analyses, including function mining, modular feature selection, sample stratification, diversity analysis, and phenotype mapping [17]. The limma-batch-effect-corrected gene expression data were used as input data. For these serial analyses, all control animals were combined into one group.

### 2.11. qRT-PCR

The expression levels of the Arntl, Cdkn1a, Per3 Slc41a3, and Bhlhe41 transcripts were measured by quantitative real-time polymerase chain reaction (qRT-PCR). Briefly, total cellular RNA was isolated from mouse heart tissues across all samples using the miRNeasy Mini Kit (Qiagen), as previously described. The High-Capacity cDNA Reverse Transcription Kit (Applied Biosystems, 4368814) was used to generate cDNA according to the manufacturer’s instructions. Quantitative RT-PCR was performed using an Applied Biosystems 7700 apparatus. The relative expression levels of the target gene mRNAs were calculated by the comparative C_T_ method. Differences in C_T_ values were calculated for each target mRNA after subtracting the mean value of 18S rRNA (relative expression = 2^−ΔCT^) [19]. Data were normalized using 18S ribosomal RNA (rRNA). Forward and reverse primer sequences are provided in Table 2.

### 2.12. Gene Enrichment Analyses

After the identification of the 12 common regulated genes (Spon2, Adam19, Arntl, Cdkn1a, Cry2, Per2, Per3, Wee1, Rcan1, Slc41a3, Errfi1, and Bhlhe41) across all 3 radiation types (^137^Cs, ^14^Si, and ^22^Ti), gene enrichment analysis was repeated using the ENRICHR resource (http://amp.pharm.mssm.edu/Enrichr/, accessed 09/2020), an integrative web-based gene list enrichment analysis tool that includes the 2019 Kyoto Encyclopedia of Genes and Genomes (KEGG) Mouse database and the ChEA 2016 gene library for identification of enriched terms and upstream transcription factors, respectively. The clustergrammer shows heatmaps of enriched terms as columns.

### 2.13. Statistical Analysis of qPCR Results

Data are presented as the mean ± standard error of the mean (SEM). mRNA expression levels of Arntl, Cdkn1a, Per3, Slc41a3, and Bhlhe41 between non-exposed and exposed animals were analyzed using one-way analysis of variance with the Bonferroni correction for comparisons between >2 groups. Statistical analysis was performed using GraphPad Prism 6, version 6.07 (GraphPad Software, Inc., La Jolla, CA, USA). Differences were considered statistically significant at *p* < 0.05.

## 3. Results

### 3.1. Data Collection, Pre-Processing, and Mapping

Since samples were processed for microarray analyses in two separate batches, we first performed a batch effect assessment. PCA clearly showed that samples were divided into two clusters. To minimize the observed effects on gene expression values, we performed batch effect removal using methods described in the Batch Examination and Correction section of Materials and Methods. Assessment of batch-related effects with principal variance component analysis showed the best adjustment and was done by limma (Supplementary Figure S1A–E), and these data were used for differential gene expression analysis.

Next, we performed a portrayal of transcriptome landscapes for studied groups using the SOM approach (Supplementary Figure S2). This method projects gene expression data onto a two-dimensional grid of 40 × 40 pixels (SOM portraits) and allows for visualization of clusters of up- and downregulated genes as spot-like structures on a map of red or blue color, respectively. Inspection of deregulated spot distribution on the group-centered SOM portraits demonstrated that radiation exposure triggers variations in the transcriptome landscape compared to untreated animals. Moreover, it could be noted that the highest variability of gene expression changes is associated with the particle type (Supplementary Figures S2 and S3). Less pronounced yet visible differences in transcriptome landscapes were also associated with particle exposure dosage. As these results warrant further exploration, we performed a detailed analysis of DEGs and functional annotation analysis.

### 3.2. Different Gene Expression in Mouse Heart Tissue Following ^137^Cs Irradiation

**Figure 1:**
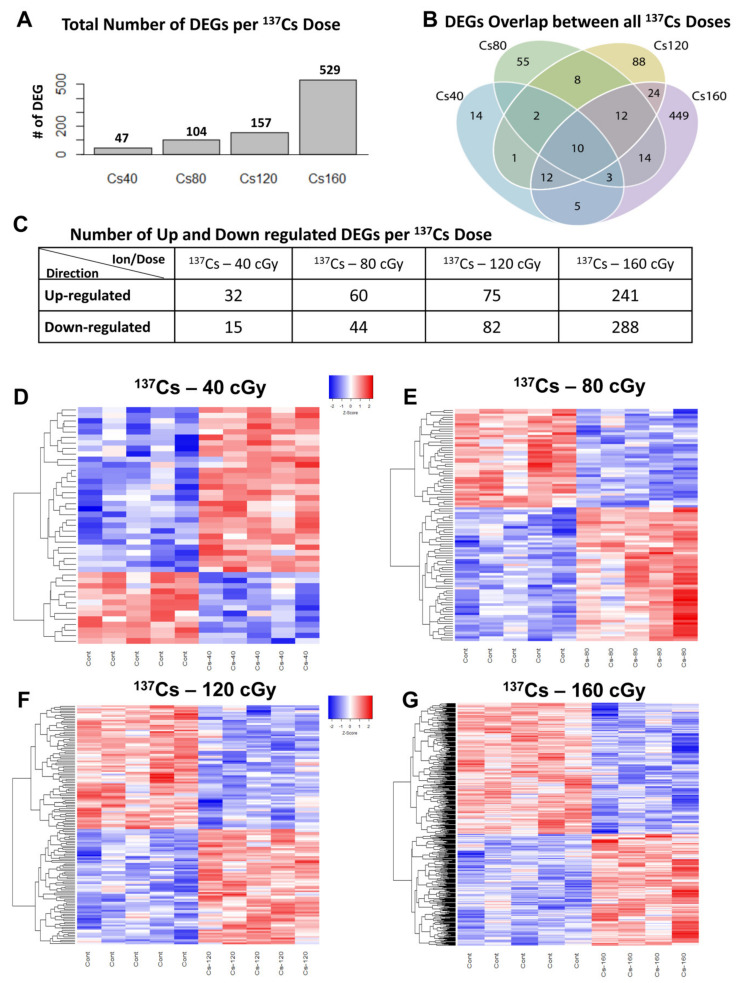
Different gene expression in mouse heart tissue following ^137^Cs-gamma irradiation: (**A**) bar graphs showing differentially expressed genes in the mouse heart tissue at different ^137^Cs (Cs, cesium) doses and (**B**) Venn diagram depicting the number of differentially expressed genes in the mouse hearts across different doses of ^137^Cs irradiation. Colors in the diagram represent genes putatively affected by the following ^137^Cs dose exposure: blue circles correspond to 40 cGy, green circles to 80 cGy, yellow circles to 120 cGy, and purple circles to 160 cGy. (**C**) Table depicting the number of up- and downregulated genes at different ^137^Cs-IR doses in the heart tissue and (**D**–**G**) heat maps showing differentially expressed genes at different ^137^Cs-IR doses. Relative expression values are indicated on the color key and increase in value from blue to red (*n* = 5/group).

We evaluated DEGs in mouse hearts exposed to different doses of ^137^Cs-IR (Figure 1A–G). The analysis revealed changes in DEGs in particle-exposed animals vs. controls (adjusted *p* < 0.25). We identified a clear dose-dependent increase in the number of DEGs in ^137^Cs-IR-exposed mouse hearts. The highest number of DEGs (529 genes, 241 upregulated and 288 downregulated) was observed after the highest dose, 160 cGy ^137^Cs-IR (Figure 1A–C,G and Supplementary Spreadsheet_Cs_DEG_Up_Down). Conversely, the lowest number of DEGs was seen in 40 cGy ^137^Cs-IR-exposed mouse hearts, where we found 47 DEGs, of which 32 were upregulated and 15 downregulated (Figure 1A–D and Supplementary Table S3). After 80 cGy ^137^Cs-IR, we identified 104 DEGs, of which 60 were upregulated and 44 downregulated (Figure 1A–C,E and Supplementary Table S3). After 120 cGy ^137^Cs-IR, we found 157 DEGs, of which 75 were upregulated and 82 downregulated (Figure 1A–C,F and Supplementary Table S3). Overall, 10 overlapping genes were common for all four doses of ^137^Cs-IR (Figure 1B). Of these 10 genes, 8 were upregulated (Dbp, Gstt2, Pik3ip1, Tspan4, Tcap, Stc2, P2rx7, and Nr1d2) and 2 were downregulated (Errfi1 and Arntl). These 10 common genes are implicated in the modulation of circadian rhythms [20,21,22], regulation of muscle cell differentiation and locomotor activity [23], cardiac hypertrophy [24], metabolism [23,25,26], oxidative stress [27,28,29], inflammation [30,31,32], and tumorigenesis [23,33,34,35,36].

### 3.3. Different Gene Expression in Mouse Heart Tissue Following ^14^Si Irradiation

**Figure 2:**
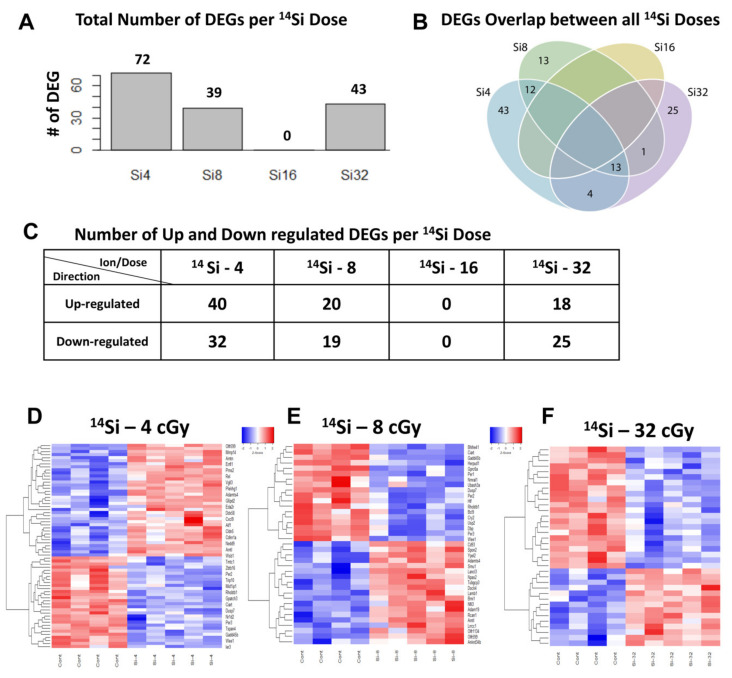
Different gene expression in mouse heart tissue following ^14^Si irradiation: (**A**) bar graphs showing differentially expressed genes in mouse heart tissue after exposure to different ^14^Si (Si, silicon) doses and (**B**) Venn diagram depicting the number of differentially expressed genes in mouse hearts across different doses of ^14^Si irradiation exposure. Colors in the diagram represent genes putatively affected by the following ^14^Si dose exposure: blue circles correspond to 4 cGy, green circles to 8 cGy, yellow circles to 16 cGy, and purple circles to 32 cGy. (**C**) Table depicting the number of up- and downregulated genes at different ^14^Si-IR doses in the heart tissue and (**D**–**F**) heat maps showing differentially expressed genes at different ^14^Si-IR oses. Relative expression values are indicated on the color key and increase in value from blue to red (*n* = 5/group).

Next, we evaluated the gene expression changes in mouse hearts subjected to ^14^Si-IR (260 MeV/n) exposure at varying doses. The analysis revealed significant changes in DEGs (adjusted *p* < 0.25). Notably, the highest number of DEGs (72 genes, 40 upregulated and 32 downregulated) was observed in ^14^Si-IR-exposed mice after the lowest dose of 4 cGy (Figure 2A–D and Supplementary Table S4), with no overlapping genes between any of the varying ^14^Si-IR doses (Figure 2B). In mouse hearts exposed to 8 cGy ^14^Si-IR, we found 39 DEGs, of which 20 were upregulated and 19 downregulated (Figure 2A–C,E and Supplementary Table S4). Surprisingly, we did not find any DEGs in the 16 cGy ^14^Si-IR groups (Figure 2A–C), whereas after 32 cGy of ^14^Si-IR, we detected 43 DEGs, of which 18 were upregulated and 25 downregulated (Figure 2A–C,F and and Supplementary Table S4).

### 3.4. Different Gene Expression in Mouse Heart Tissue Following ^22^Ti Irradiation

**Figure 3:**
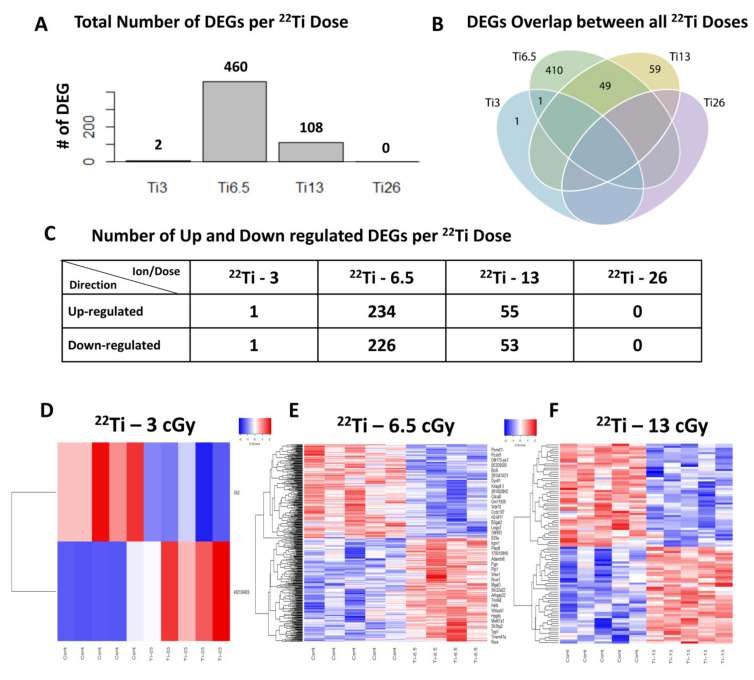
Different gene expression in mouse heart tissue following ^22^Ti irradiation: (**A**) bar graphs showing differentially expressed genes in mouse heart tissue after exposure to different ^22^Ti (Ti, titanium)-IR exposure. Colors in the diagram represent genes putatively affected by the following ^22^Ti dose exposure: blue circles correspond to 3 cGy, green circles to 6.5 cGy, yellow circles to 13 cGy, and purple circles to 26 cGy. (**B**) Venn diagram depicting the number of differentially expressed genes in mouse hearts across different doses of Ti radiation exposure. Colors in the diagram represent genes putatively affected by the following Ti dose exposure: blue circles correspond to 3 cGy, green circles to 6.5 cGy, yellow circles to 13 cGy, and purple circles to 26 cGy. (**C**) Table depicting the number of up- and downregulated genes after exposure to different ^22^Ti-IR doses in the heart tissue and (**D**–**F**) heat maps showing differentially expressed genes at different ^22^Ti-IR doses. Relative expression values are indicated on the color key and increase in value from blue to red (*n* = 5/group).

Further analysis of DEGs in mouse hearts subjected to different doses of ^22^Ti-IR (1000 MeV/n) revealed significant changes in DEGs (adjusted *p* < 0.25). The highest number of DEGs (460 genes, 234 upregulated and 226 downregulated) was observed after a very low dose of 6.5 cGy ^22^Ti-IR (Figure 3A–C,E and and Supplementary Table S5). We identified 49 overlapping DEGs between the 6.5 and 13 cGy ^22^Ti-IR groups (Figure 3B). At the lowest dose of 3 cGy ^22^Ti-IR, we found only 2 DEGs, of which 1 was upregulated and 1 downregulated (Figure 3A–D and and Supplementary Table S5). In the hearts of 13 cGy ^22^Ti-IR-exposed mice, we found 108 DEGs, of which 55 were upregulated and 53 downregulated (Figure 3A–C,F and Supplementary Table S5). Surprisingly, we did not detect any DEGs with ≥twofold change in mouse hearts subjected to the highest dose of 26 cGy ^22^Ti-IR (Figure 3A–C). Similar to our previous findings after different doses of ^14^Si-IR, we did not find any overlapping DEGs between the four ^22^Ti-IR doses (Figure 2B and Figure 3B).

### 3.5. Common Differentially Expressed Genes across All Radiation Types

**Figure 4:**
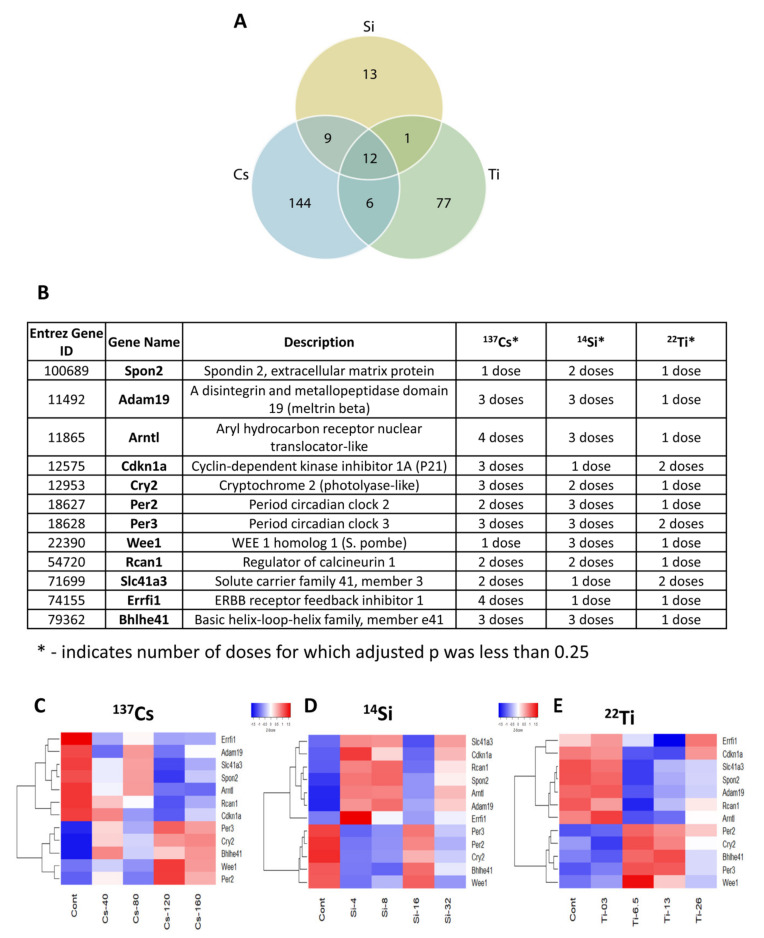
Overlapping differentially expressed genes: (**A**) Venn diagram depicting the overlap of enrichment terms amongst three different radiation exposure groups (^137^Cs, ^14^Si, and ^22^Ti) compared to the control group. In the diagram, blue circles represent genes putatively affected by long-term ^137^Cs exposure, yellow circles represent genes influenced by ^14^Si exposure, and green circles represent those affected by ^22^Ti exposure. (**B**) Table depicting 12 commonly expressed genes in one or more of all radiation-type exposures and (**C**–**E**) microarray data analysis showing heat maps of the 12 overlapping commonly expressed genes in different radiation groups/doses. Relative expression values are indicated on the color key and increase in value from blue to red (*n* = 5/group).

We identified 12 overlapping genes that were common for all three types of IR and were detected in at least one or more IR doses for each ion (Figure 4A–E). These 12 overlapping genes are associated with a variety of biological and physiological processes, including food intake, body weight, sleep alterations, circadian rhythms, inhibition of angiogenesis and myogenesis, locomotor activity, metabolism, gluconeogenesis, and lipogenesis behavior, as well as susceptibility to chronic diseases such as hypertension, diabetes, obesity, infection, and cancers of different organs. With regard to their subcellular localization, the majority of the 12 common DEGs are distributed in the nucleus (83%), cytosol (42%), plasma membrane (25%), and extracellular (47%) (Table 3). These genes are associated with the regulation of many processes within the organelles, including kinase binding (Cdkn1a, Cry2, Errfi1, Per2, Per3), transcription regulating activities (Arntl, Bhlhe41, Cry2, Per2, Spon2), histone deacetylase binding (Bhlhe41, Per2), SH3 domain binding (Adam19, Errfi1), nuclear hormone receptor binding (Cry2, Per2), protein kinase activity (Wee1), and cation transmembrane transporters (Slc41a3) (Table 4). The top intracellular pathways of the 12 overlapping genes were associated with circadian rhythms and entrainment (Arntl, Cry2, Per2, Per3, Bhlhe41), the cell cycle (Cdkn1a, Wee1), the oxytocin signaling pathway (Cdkn1a, Rcan1), and transcriptional misregulation in cancer (Cdkn1a, Per2) (Table 5).

### 3.6. Microarray Analysis and Validation

**Figure 5:**
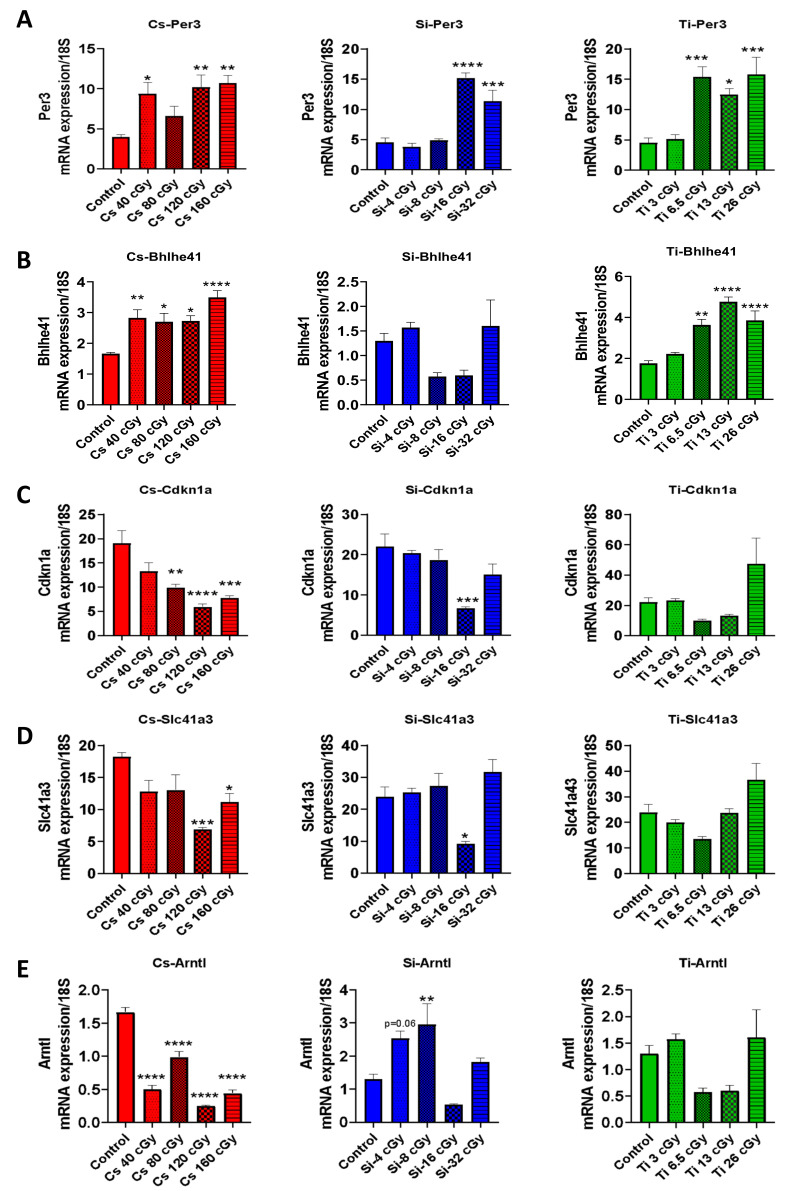
qRT-PCR validation of 5 of 12 common differentially expressed genes across all radiation types: mNA expression of (**A**) Per3, (**B**) Bhlhe41, (**C**) Cdkn1a, (**D**) Slc41a3, and (**E**) Arntl was measured by qRT-PCR in heart tissue (*n* = 3 heart/group). Data were normalized using 18S rRNA. * *p* < 0.05, ** *p* < 0.01, *** *p* < 0.001, and **** *p* < 0.0001 compared to control groups. Per3, period circadian regulator 3; Bhlhe41, basic helix-loop-helix family member E41; Cdkn1a, cyclin-dependent kinase inhibitor 1A; Arntl, aryl hydrocarbon receptor nuclear translocator like; Slc41a3, solute carrier family 41 member 3.

To validate our microarray analysis findings of the 12 overlapping DEGs across the three different radiation groups (Figure 4A–F), we tested five DEGs, namely period circadian regulator 3 (Per3), basic helix-loop-helix family member E41 (Bhlhe41), cyclin-dependent kinase inhibitor 1A (Cdkn1a), solute carrier family 41 member 3 (Slc41A3), and aryl hydrocarbon receptor nuclear translocator like (Arntl), by qRT-PCR. Our qRT-PCR analysis revealed that only Per3 was found significantly regulated in all conditions (Figure 5A–E vs. Figure 4C–E). We also found that Bhlhe41, Cdkn1a, Slc41A3, and Arntl were all significantly upregulated after ^137^Cs-IR exposure in a dose–response manner. However, only Bhlhe41 was found to be upregulated in the ^22^Ti-IR group at high doses. Our results also showed that Cdkn1 and Slc41A3 were significantly downregulated post-^16^Si-IR exposure, while Arntl was found to be significantly upregulated at lower doses (^8^Si).

### 3.7. Gene Enrichment Analysis of 12 Common DEGs

**Figure 6:**
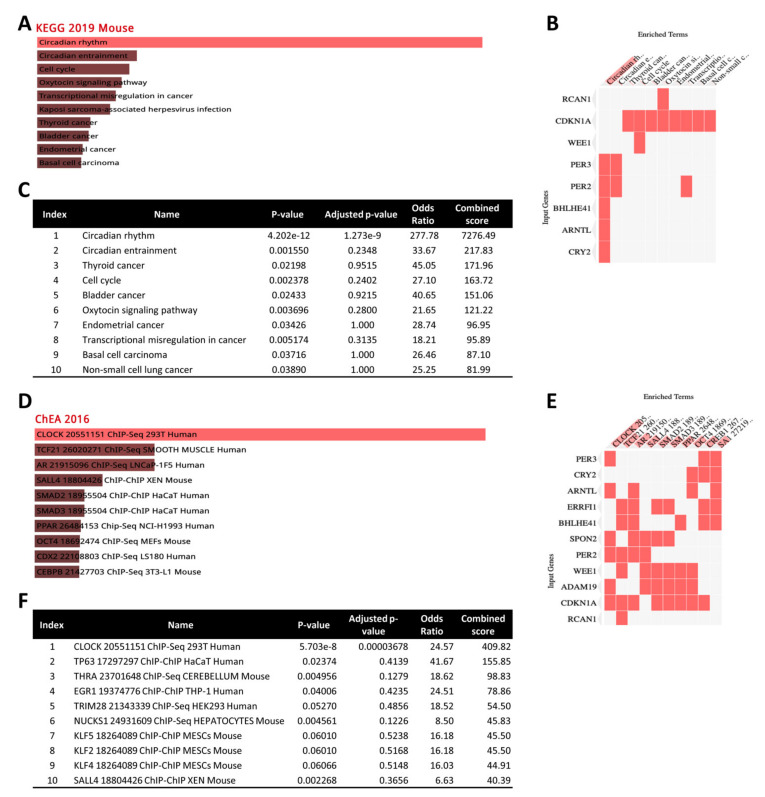
A comprehensive gene set enrichment analysis for the 12 common genes differentially regulated across all 3 radiation groups was performed using EnrichR and the Kyoto Encyclopedia of Genes and Genomes (KEGG) 2019 Mouse database and ChIP-X enrichment analysis (ChEA) gene set library. (**A**) Bar graphs (sorted by *p*-value ranking) represent the significance of that specific gene set using the KEGG 2019 Mouse database. The brighter the color, the more significant that term. (**B**) The clustergrammer is sorted by combined scores (*p*-value and *z*-score) and shows heatmaps. Enriched terms are shown as columns and input genes as rows to understand the relationships between input genes and enriched terms. Cells in the matrix indicate whether a gene is associated with the indicated term. (**C**) The table shows a raw view of the data and is sorted by *p*-value. Terms, *z*-score, odds ratio, and combined score are indicated. (**D**) Bar graphs (sorted by *p*-value ranking) represent the significance of identified upstream transcription factors using the ChEA 2016 gene set library. The brighter the color, the more significant the transcription factor (TF). (**E**) The clustergrammer is sorted by *p*-value and shows heatmaps of enriched terms as columns. Input genes are shown as rows to understand the relationships between the input genes and enriched TFs. Cells in the matrix indicate whether a gene is associated with the predicted TF. (**F**) The table shows a raw view of the data and is sorted by *p*-value. Terms, *z*-score, odds ratio, and combined score are indicated.

To further identify potentially compromised biological pathways, we performed a comprehensive gene set enrichment analysis for the 12 common genes (Spon2, Adam19, Arntl, Cdkn1a, Cry2, Per2, Per3, Wee1, Rcan1, Slc41a3, Errfi1, and Bhlhe41) across all three radiation types using Enrichr and pathway databases such as the 2019 Kyoto Encyclopedia of Genes and Genomes (KEGG) for Mouse (Figure 6). KEGG pathway enrichment analysis revealed that pathways associated with circadian rhythms, cancer (thyroid, bladder, endometrial, non-small-cell lung cancer), the cell cycle, and the oxytocin signaling pathway were the most enriched by these DEGs (Figure 6A–C). We then evaluated these DEGs against the ChIP-X enrichment analysis (ChEA) gene set library to calculate enrichment for upstream transcription factors. The ChEA 2016 database is a gene set library that contains putative targets for transcription factors, based on reports profiling transcription factors binding to DNA in mammalian cells. This analysis identified 10 transcription factors (CLOCK, TCF21, AR, SALL4, SMAD2, SMAD3, PPAR, OCT4, CDX2, and CEBPB) that may be involved in the regulation of the 12 common DEGs across the three types of radiation (Figure 6D–F).

**Figure 7:**
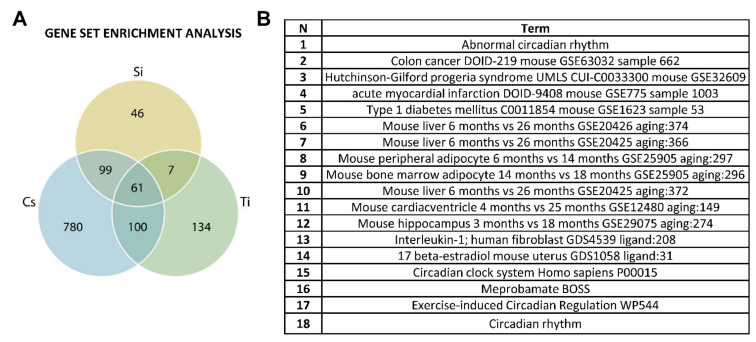
Overlapping biological processes: (**A**) Venn diagram analysis of differentially expressed genes across all three different radiation exposure groups/doses compared to controls. In each paired test, the differentially expressed genes were identified as a log2 fold change and *p* < 0.05. In the diagram, blue circles represent genes putatively affected by long-term ^137^Cs exposure, green circles represent genes influenced by ^14^Si exposure, and yellow circles represent genes that were putatively affected by ^22^Ti exposure. (**B**) The table represents various biological processes that are common for all three radiation groups.

Further enrichment analysis revealed that ^14^Si-IR exposure dysregulated 213 biological processes in the heart tissue, whereas ^137^Cs-IR exposure alone affected 1040 biological processes, and ^22^Ti-IR exposure dysregulated 302 biological processes. Interestingly, we found 61 overlapping biological processes that were common for all three radiation groups compared to controls (Figure 7A). Top hits for biological processes included abnormal circadian rhythm, colon cancer, Hutchinson–Gilford progeria syndrome, acute myocardial infarction, type 1 diabetes, etc. (Figure 7B).

Finally, we were interested in understanding the extent of the genes and associated biological processes that were shared between particle doses causing the maximal effect (^137^Cs at 160 cGy, ^14^Si at 4cGy, and ^22^Ti at 6.5 cGy). The analysis showed little overlap in terms of up- or downregulated genes (Supplemental Figure S4A–D). Four upregulated overlapping gene sets were detected in all three conditions, and more when performing pairwise comparisons (Supplemental Figure S4A–D). While non-overlapping, each pairwise comparison revealed a considerable number of heart-disease-related gene sets, suggesting that particle irradiation may affect cardiac function through various mechanisms. No common downregulated gene sets were detected (Supplementary Table S6).

### 3.8. Regression Analysis of Common Differentially Expressed Genes across Radiation Type

**Figure 8:**
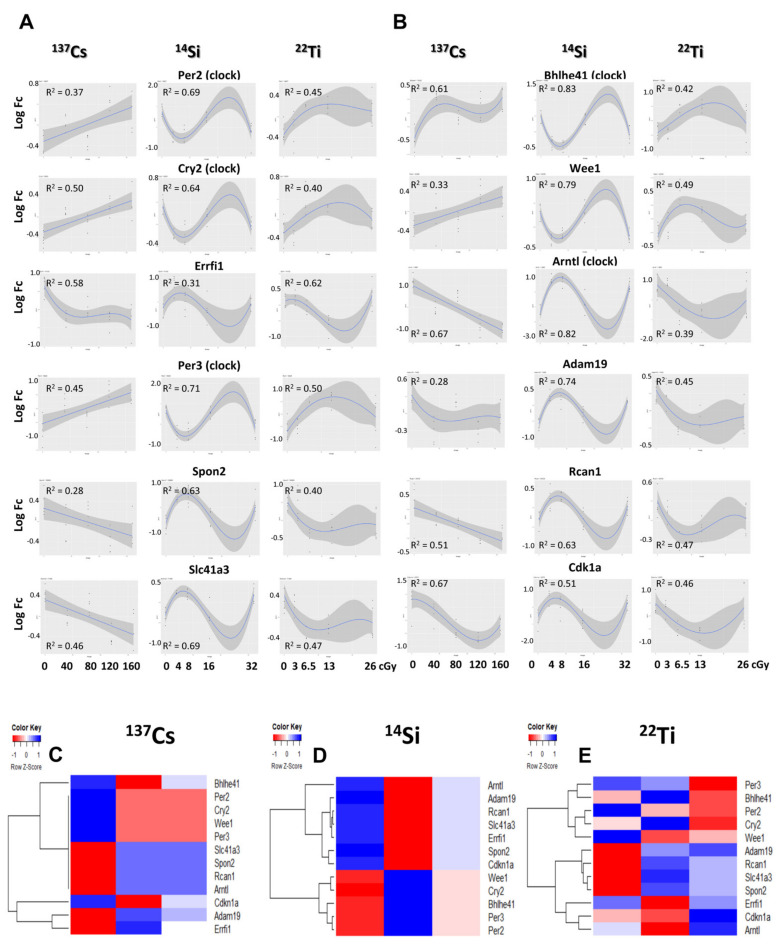
Dose–response analyses of expression of 12 genes commonly deregulated in all three radiation conditions: (**A**,**B**) expression profile–exposure dosage analysis indicates that ^137^Cs irradiation mostly causes linear changes in gene expression, while irradiation with ^14^Si and ^22^Ti mainly induces a non-linear gene expression response. (**C**–**E**) Heatmap clustering of non-linear regression fit coefficients for 12 common genes in ^137^Cs-, ^14^Si-, and ^22^Ti-IR groups, respectively. The columns in heatmaps represent non-linear fit coefficients.

Next, we assessed the 12 overlapping DEGs using dose-dependent regression analyses (see the Materials and Methods section for details). Our findings revealed an exclusive non-linear differential gene expression pattern in ^14^Si- and ^22^Ti-IR-exposed hearts, whereas two-thirds (66.7%) of ^137^Cs-IR-exposed hearts revealed a linear pattern of gene expression (Figure 8A,B and Table 6). Further, dose–response shape-based clustering confirmed the predominantly linear response in ^137^Cs-IR-exposed and non-linear responses in ^14^Si- and ^22^Ti-IR-exposed hearts (Figure 8C,D).

## 4. Discussion

We used archived mouse hearts from NASA’s Space Radiation Tissue Sharing Forum collected from non-tumor-bearing female CB6F1/Hsd mice after single whole-body irradiation with different low or very low doses of ^137^Cs-gamma, ^14^Si, or ^22^Ti-ion IR to determine the long-term effects of radiation exposure on gene expression in the whole heart as a function of IR type and dose compared to sham-treated controls. We aimed to determine whether high-LET HZE particle-IR exposure may induce biological responses at lower doses compared to ^137^Cs-gamma IR exposure. Our data revealed a dose-dependent increase in the number of DEGs (both up- and downregulated) following exposure to increasing doses of ^137^Cs-IR, while no discernable pattern of DEGs was observed in hearts irradiated with ^14^Si or ^22^Ti-ion. In contrast to ^137^Cs-gamma IR, where the largest number of DEGs was observed after the highest dose (160 cGy), the largest number of DEGs was observed at the lowest dose of ^14^Si-IR (4 cGy) and the second-lowest dose of ^22^Ti-IR (6.5 cGy), suggesting that compared to ^137^Cs-gamma IR, even low doses of HZE-IR may be sufficient to induce long-term dysregulation of genes in the heart tissue. In the ^14^Si-ion IR group (260 MeV/n), no DEGs were detected at an intermediate dose of 16 cGy but were detected at the higher dose of 32 cGy, while the lowest number or absence of DEGs was observed at minimum and maximum doses of ^22^Ti-ion IR (3 and 26 cGy, 1 GeV/n). It is possible the effects of these radiation types and respective doses may be too subtle for the detection of DEGs based on statistical criteria or may have resulted in extensive damage of a few heavily damaged cells at the cellular level, resulting in early death and clearance of the affected cells without long-term consequences on the gene expression in the whole tissue. Considering that low doses of HZE-ion IR may be sufficient to induce changes in genes involved in disease pathophysiology, further studies are needed to better delineate the effects of these radiation types and doses, along with the mechanisms triggering these varied responses, in order to develop better assessments of radiobiological effectiveness.

Among all groups, irrespective of radiation type or dose, no clear lower IR threshold for gene expression was detected. However, the gene signatures were indicative of varying cardiovascular diseases such as hypertension, atherosclerosis, cardiomyopathies (non-ischemic, dilated, peripartum, hypertrophic), heart failure, acute myocardial injury, and myocarditis, as well as known cardiovascular risk factors, including but not limited to obstructive sleep apnea, idiopathic lung fibrosis, diabetes, and obesity. It is therefore of utmost importance to further investigate the disrupted pathways and linked gene networks to determine the presence of early biomarkers for disease risk and/or inform the development of mitigating factors for radiation-induced cardiovascular and other degenerative diseases during and after exploration-type spaceflight missions.

We identified 12 genes differentially regulated at at least one or more doses of all three radiation types. These include Spon2, Adam19, Arntl, Cdkn1a, Cry2, Per2, Per3, Wee1, Rcan1, Slc41a3, Errfi1, and Bhlhe41, which are related to numerous cardiovascular and degenerative diseases of other organs as well as varying aging processes. For example, Spon2 encodes mindin, an extracellular matrix protein highly expressed in cardiac tissue, and plays various roles, including (1) serving as a ligand for integrin during inflammation [37], (2) inhibiting angiogenesis [38], and (3) playing an anti-hypertrophic role, protecting against cardiac remodeling observed in heart failure by attenuating AKT/GSK3β and TGF-β1-Smad signaling [39]. We also found cyclin-dependent kinase inhibitor 1A (Cdkn1A), also known as p21, to be dysregulated with different radiation types/doses, which correlates with previously published reports [40]. P21 is a well-known potent universal CDK inhibitor [41] and regulates diverse biological functions such as cell growth, proliferation, apoptosis, and inflammation [42,43]. It has been primarily described as a major regulator of p53-dependent cell cycle arrest in response to DNA damage. Previous aging studies reported that p21-knockout mice were more susceptible to developing spontaneous tumors and exhibited early tumor onset and more severe atherosclerotic lesions compared to wild-type mice [44,45].

Interestingly, among the 12 identified overlapping genes, 5 are directly related to the regulation of circadian rhythms: Per2, Per3, Cry2, Bhlhe41, and Arntl [46]. In addition to these circadian-rhythm-associated genes, we also identified the upstream transcription factor CLOCK [47], predicted to be upregulated across all radiation types/doses. The fundamental role of circadian rhythms is the diurnal regulation of behavior and physiological processes over 24 h, and they are of interest in spaceflight, considering the entrainment signals of light/dark are disrupted during low-Earth-orbit (LEO) flights [48] and are expected to be disrupted during future exploration-type space missions. Recent studies have highlighted the critical role of circadian rhythms in regulating cardiac metabolism, function, and response to injury [49].

The diurnal regulation of circadian rhythms involves feedback loops of transcription and translation. The CLOCK gene has DNA-binding histone acetyltransferase activity. It plays a critical role in regulating circadian rhythms by forming heterodimers with other clock proteins such as Arntl and promoting transcription of period (Per1, Per2, and Per3) [50] and cryptochrome (Cry1, Cry2) genes [46]. Per2 has been shown to play a cardioprotective role in myocardial ischemia. Its dysregulation is associated with increased cardiovascular disease risk as a result of impaired metabolic pathways, including fatty acid metabolism, cardiac anaerobic glycolysis, other pathways involved in vascular senescence and endothelial dysfunction, and dysregulation of cardiac ion channels, which play critical roles in the development of arrhythmias [51,52,53,54,55]. Such dysregulation may also result in the development of endothelial and vascular phenotypes observed in cardiovascular disease. Per3 is involved in delayed sleep-phase syndrome and cardiac autonomic control during sleep [56]. Considering its multifaceted regulatory roles, the disruption of circadian rhythms can potentially play a large role in many disease processes associated with low-dose space-type radiation.

We observed downregulation of Arntl with increasing doses of ^137^Cs-gamma IR, whereas ^14^Si-ion IR exposure up to 8 cGy significantly upregulated Artnl. ^22^Ti-ion IR did have a significant effect on Arntl expression at any dose. Differences in the radiation quality may be important to this observation as the silicon ions were 70 keV/mm, and the titanium were 100 keV/mm with greater ionization densities. Altered levels of Arntl have been associated with increased susceptibility to hypertension, diabetes, obesity, and defective lipogenesis [57,58]. Per3 expression dose-dependently followed ^137^Cs-gamma IR, whereas higher doses of ^14^Si- ion IR (16 and 32 cGy) and ^22^Ti-ion IR (greater than 6.5 cGy) exhibited increased Per3 expression. Furthermore, defects in Bhlhe41, a transcription factor repressor in circadian rhythms, are associated with altered sleep patterns [59] and are reported to play a significant role in immune function [60], as well as epithelial-to-mesenchymal transition [61]. Bhlhe41 expression was elevated at all ^137^Cs-gamma IR doses, whereas no effect on expression was noted across all doses of ^14^Si-ion IR. Higher doses of ^22^Ti-ion IR (6.5–26 cGy) significantly increased Bhlhe41 expression. The differential regulation of CLOCK genes can disrupt components downstream to the circadian cycle regulatory mechanism. Thus, regulation of the circadian rhythm genes may represent a potential mitigation mechanism for the well-being of astronauts during long-duration space missions [62].

In addition to CLOCK, our gene enrichment analysis identified nine other transcriptions factors (TCF21, AR, SLAL4, SMAD2, SMAD3, OOAR, OCT4, CDX2, and CEBPB), which are implicated in various cellular processes, including metabolism [63,64], inflammation [65,66,67], rhythmic activation of circadian cycle repressors [68], and maintenance of the pluripotency of stem cells [69,70,71,72]. Many of these factors act as key mediators of canonical signaling pathways such as SMAD2 and SMAD3, involved in TGF-β signaling. They, thus, are involved in cell proliferation, apoptosis, and differentiation [73,74,75,76]. Additionally, many play important roles in tumor processes, including tumor-associated angiogenesis and induction of epithelial-to-mesenchymal cell transitioning [77,78,79,80,81,82]. Thus, there are many potential avenues to explore to understand and mitigate the cardiovascular response in the setting of radiation exposure during spaceflight.

There are few limitations worth mentioning. First, we used a relaxed cutoff threshold (adjusted *p* < 0.25), which could include more false positives compared to a stricter cutoff. In contrast, it is well known that a small sample size generally yields higher adjusted *p*-values [83,84,85,86]. However, the results of downstream functional analyses and data from previous publications further confirmed the validity of our methodology and findings.

## 5. Conclusions

In this study, we demonstrated that exposure to ionizing radiation causes a change in the transcriptomic profile of hearts from whole-body-irradiated mouse hearts that is dependent on the quality and quantity of radiation. The effects of low-LET ^137^Cs-gamma rays and high-LET ^14^Si- and ^22^Ti-ion IR are distinctly different, yet none presented a clear lower IR threshold for differential gene expression in this study. Dose-dependent regression analyses of the 12 identified differentially expressed common genes for all three radiation types showed a dose-dependent linear response in 67% (8/12) of the genes following ^137^Cs-gamma IR, consistent with changes induced in the whole transcriptomic profile after this type of IR exposure. At the same time, there was an exclusive non-linear DEG pattern with ^14^Si- or ^22^Ti-ion IR. Moreover, HZE-ion IR-exposed hearts showed the largest change in gene expression at lower doses, indicating a greater biological response at low and very low doses, in part, due to the different quality of HZE-ion IR-induced tissue damage. Multiple genes involved in cardiovascular and other degenerative diseases were attenuated with exposure to all three radiation types. Overall, the data in this manuscript points to metrics in the form of differential gene expression that could be used in assessing cardiovascular risks astronauts face during deep-space missions but also risks for other neurodegenerative diseases, aging processes, and cancers of various organs. However, future mechanistic and functional studies, including acute, intermediate, and late time points using additional lower doses to determine a possible lower threshold, would give us a comprehensive understanding of HZE-ion IR’s effects on human health and disease.

## Figures and Tables

**Table 1:** Ionizing radiation types. Beam energies, doses, and LET used in this study.

Ion	Dose (cGy)	Energy (MeV/n)	Entrance LET (keV/µ)
**^137^Cs**	0 40	0.662	0.8
	80		
	120		
	160		
**^14^Si**	0 4	260	70
	8		
	16		
	32		
**^22^Ti**	0 3	1000	100
	6.5		
	13		
	26		

**Table 2:** Quantitative RT-PCR primers used in this study.

Gene	Primer
**ARNTL**	Probe-/56-FAM/ACC TGC TCC/ZEN/AGT GTT TCC TCA TCA/3IABkFQ/
	R-ATC CAC AGC TAG CCC AAA C
	F-CCA CCT CAG AGC CAT TGA TAC
**Slc41a3**	Probe-/56-FAM/CTG GTT TCT/ZEN/GTG CCT CCC TGA CTG/3IABkFQ/
	R-CAC TGA GGA CAT GAG GGA AAG
	F-CTT CTT CCT GGA CTG GTT ACT G
**Cdkn1a**	Probe-/56-FAM/CAG CCT AGA/ZEN/ACA GGG ATG GCA GTT/3IABkFQ/
	R-GAG TCG GGA TAT TAC GGT TGA G
	F-CCA GCT AGG ATG ACA GTG AAG
**PER3**	Probe-/56-FAM/AGC CGG AAG/ZEN/GTC TCC TTC ATC ATT/3IABkFQ/
	R-TGG ACT CGT TCG GAC TTT ATG
	F-GCA CTC AGA ACG GAG AGT ATG
**Bhlhe41**	Probe-/56-FAM/AAC CGG AAG /ZEN/CCA CAG CTC ATA CAT/3IABkFQ/
	R-TGC CTG ACT TTC TTC CCT TAC
	F-TGA CTG TGA CAA GCT GAC TG

**Table 3:** Cellular localization of 12 overlapping differentially expressed genes.

Gene Name	Extracellular	Plasma Membrane	Nucleus	Cytosol	Human Protein Atlas
**Spon2**	Y				Plasma proteins, predicted membrane proteins, predicted secreted proteins
**Adam19**		Y	Y		Plasma proteins, predicted membrane proteins
**Arntl**			Y		predicted intracellular proteins, transcription factors
**Cdkn1a**			Y	Y	Cancer-related genes, predicted intracellular proteins
**Cry2**	Y		Y	Y	Predicted intracellular proteins
**Per2**			Y		Disease-related genes, predicted intracellular proteins
**Per3**			Y	Y	Disease-related genes, predicted intracellular proteins
**Wee1**			Y		Cancer-related genes, enzymes, plasma proteins, Predicted intracellular proteins
**Rcan1**			Y	Y	Predicted intracellular proteins
**Slc41a3**		Y			Predicted membrane proteins, transporters
**Errfi1**		Y	Y	Y	Predicted intracellular proteins
**Bhlhe41**			Y		Predicted intracellular proteins, transcription factors

**Table 4:** Molecular signaling activities of 12 overlapping differentially expressed genes.

Term Description	Genes
RNA polymerase II-specific DNA-binding transcription factor binding	**Arntl, Bhlhe41, Cry2, Per2**
Kinase binding	**Cdkn1a, Cry2, Errfi1, Per2, Per3**
Transcription regulatory region sequence-specific DNA binding	**Arntl, Bhlhe41, Cry2, Per2**
Histone deacetylase binding	**Bhlhe41, Per2**
SH3 domain binding	**Adam19, Errfi1**
Nuclear hormone receptor binding	**Cry2, Per2**
Transcription regulator activity	**Arntl, Bhlhe41, Cry2, Per2**
Signaling receptor binding	**Arntl, Cry2, Per2, Spon2**
Protein kinase activity	**Wee1**
Nucleic acid binding	**Rcan1**
Cation transmembrane transporter activity	**Slc41a3**

**Table 5:** Intracellular pathways involvement of 12 overlapping differentially expressed genes.

	Spon2	Adam19	Arntl	Cdkn1a	Cry2	Per2	Per3	Wee1	Rcan1	Slc41a3	Errfi1	Bhlhe41
Circadian rhythm			+		+	+	+					+
ErbB signaling pathway				+								
HIF-1 signaling pathway				+								
FoxO signaling pathway				+								
Cell cycle, circadian regulated				+				+				
p53 signaling pathway				+								
PI3K-Akt signaling pathway				+								
Cellular senescence				+								
JAK-STAT signaling pathway				+								
Oxytocin signaling pathway				+					+			
Transcriptional misregulation in cancer				+		+						
Circadian entrainment						+	+					
Acute myeloid leukemia						+						
Human Immuno-deficiency virus 1 infection								+				
Thyroid hormone signaling pathway									+			

**Table 6:** Regression analyses of differentially expressed genes.

Icons		^137^Cs	^14^Si	^22^Ti
**Type of Regression**	Linear Regression	423	0	1
	Non-Linear Regression	215	53	123

## Data Availability

The data presented in this study are openly available in Gene Expression Omnibus (GEO), with the accession number GSE164234.

## Supplementary Materials

The following are available online at https://www.mdpi.com/2073-4409/10/2/387/s1: Supplementary Figure S1: Comparison and selection of batch adjustment methods for microarray analysis; Supplementary Figure S2: Mean irradiation exposure-related transcriptome portraits; Supplementary Figure S3: PCA of batch-adjusted gene expression matrix; Supplementary Figure S4: Overlapping DEGs and functional terms between particle doses causing the maximal effect (^137^Cs at 160 cGy, ^14^Si at 4cGy, and ^22^Ti at 6.5 cGy); Supplementary Table S1; Supplementary Table S2; Supplementary Table S3; Supplementary Table S4; Supplementary Table S5; and Supplementary Table S6.
