# Supplementary material for: Long-Term Effects of Very Low Dose Particle Radiation on Gene Expression in the Heart: Degenerative Disease Risks
Source: Cells. 2021 Feb 13;10(2):387. doi: 10.3390/cells10020387 (PMC7917872; doi:10.3390/cells10020387)
Supplement: Supplementary file 1 [file cells-10-00387-s001.zip › DGoukassian_TissueSharing_Supplemental Figures.pptx]

## Slide 1
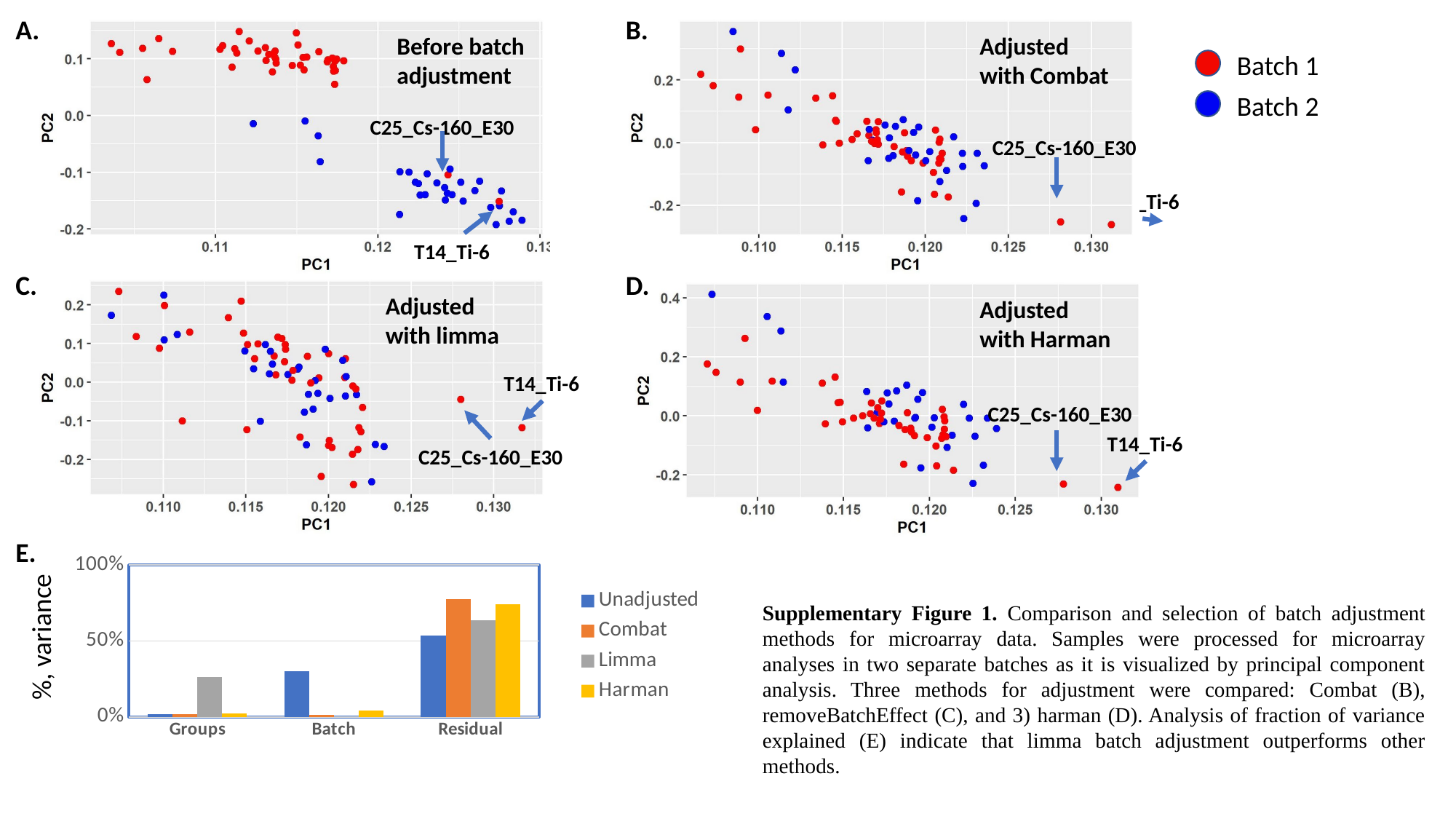

A.
B.
Before batch adjustment
C25_Cs-160_E30
T14_Ti-6
Adjusted with Combat
C25_Cs-160_E30
Batch 1
Batch 2
T14_Ti-6
C.
D.
Adjusted with limma
T14_Ti-6
C25_Cs-160_E30
Adjusted with Harman
C25_Cs-160_E30
T14_Ti-6
E.
### Chart
| Category | Unadjusted | Combat | Limma | Harman |
|---|---|---|---|---|
| Groups | 0.0184061 | 0.01756375 | 0.2617645 | 0.02146632 |
| Batch | 0.30175914 | 0.0147065 | 0.0 | 0.04151863 |
| Residual | 0.53798049 | 0.77487594 | 0.6351822 | 0.74149931 |Supplementary Figure 1. Comparison and selection of batch adjustment methods for microarray data. Samples were processed for microarray analyses in two separate batches as it is visualized by principal component analysis. Three methods for adjustment were compared: Combat (B), removeBatchEffect (C), and 3) harman (D). Analysis of fraction of variance explained (E) indicate that limma batch adjustment outperforms other methods.
%, variance

## Slide 2
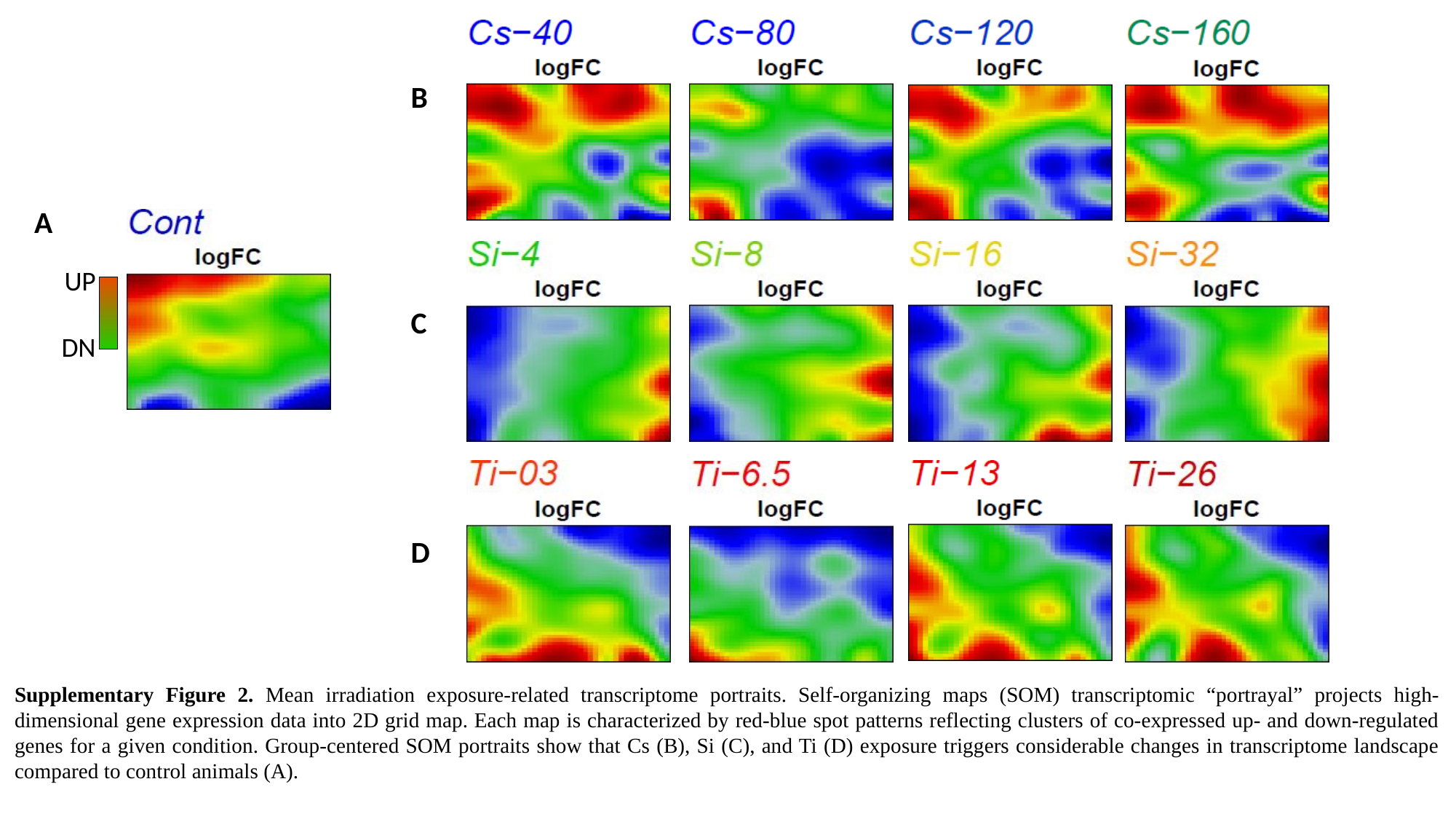

B
A
UP
C
DN
D
Supplementary Figure 2. Mean irradiation exposure-related transcriptome portraits. Self-organizing maps (SOM) transcriptomic “portrayal” projects high-dimensional gene expression data into 2D grid map. Each map is characterized by red-blue spot patterns reflecting clusters of co-expressed up- and down-regulated genes for a given condition. Group-centered SOM portraits show that Cs (B), Si (C), and Ti (D) exposure triggers considerable changes in transcriptome landscape compared to control animals (A).

## Slide 3
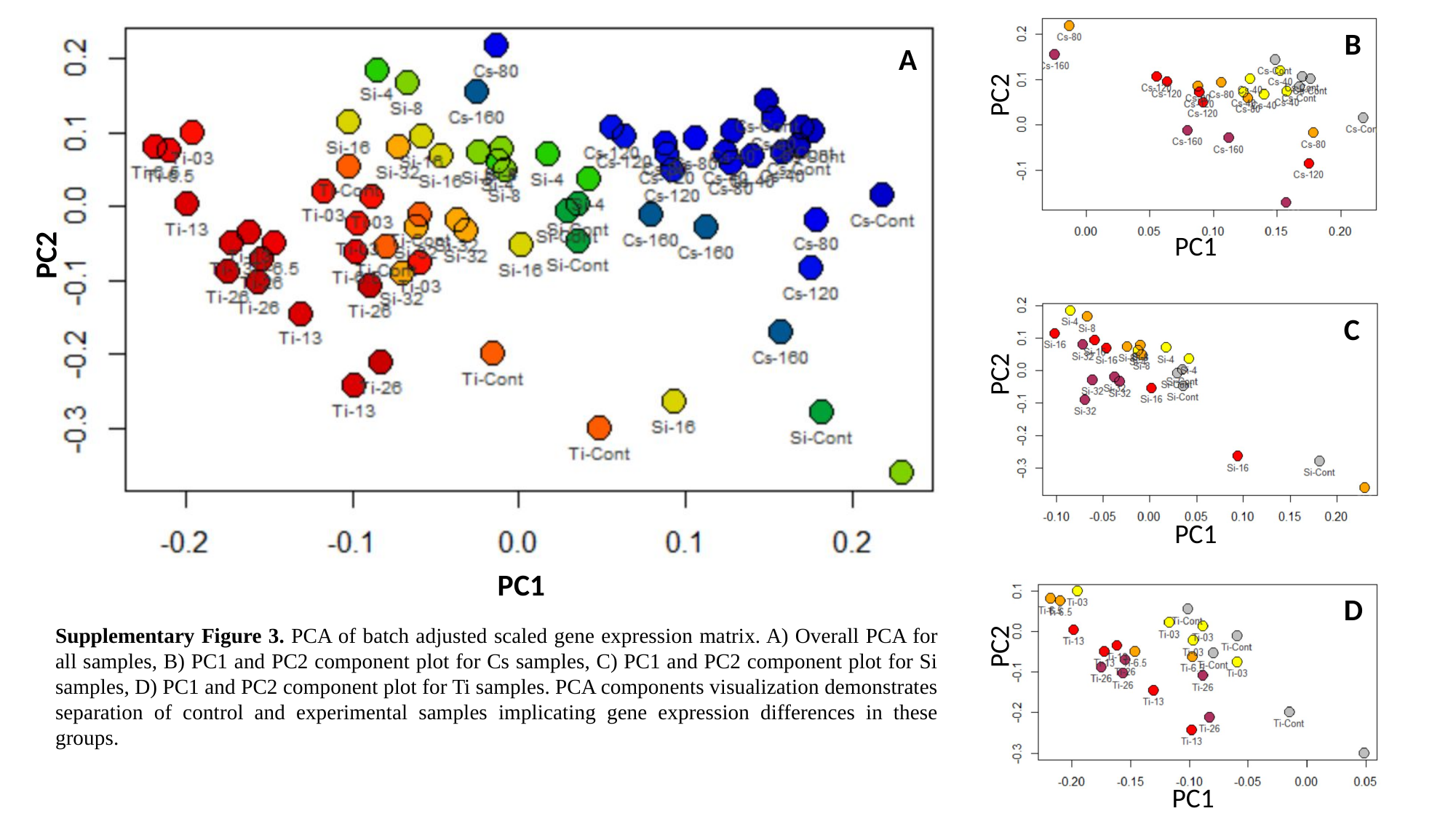

B
A
PC2
PC1
PC2
C
PC2
PC1
PC1
D
Supplementary Figure 3. PCA of batch adjusted scaled gene expression matrix. A) Overall PCA for all samples, B) PC1 and PC2 component plot for Cs samples, C) PC1 and PC2 component plot for Si samples, D) PC1 and PC2 component plot for Ti samples. PCA components visualization demonstrates separation of control and experimental samples implicating gene expression differences in these groups.
PC2
PC1

## Slide 4
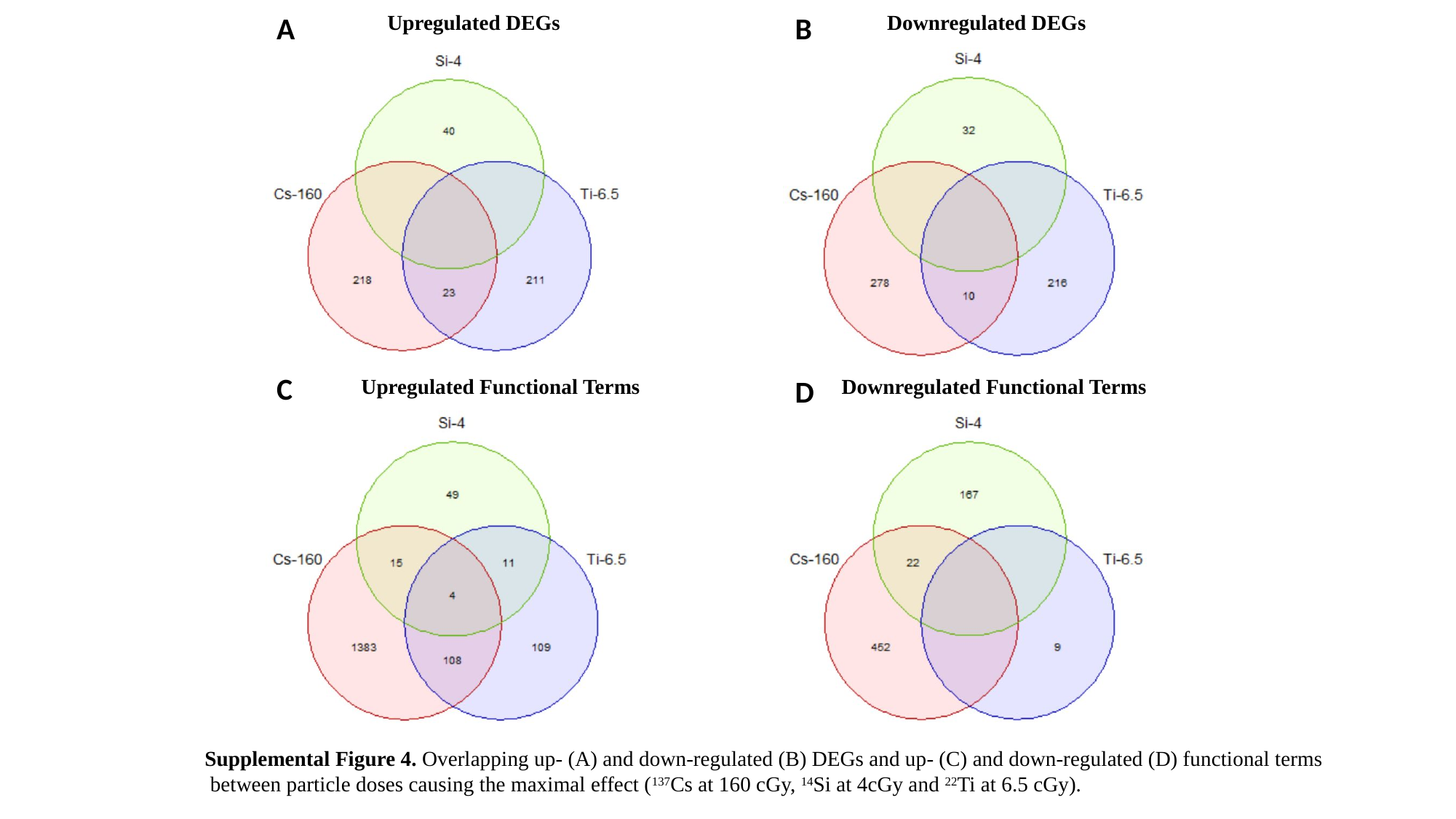

A
Upregulated DEGs
B
Downregulated DEGs
C
Upregulated Functional Terms
Downregulated Functional Terms
D
Supplemental Figure 4. Overlapping up- (A) and down-regulated (B) DEGs and up- (C) and down-regulated (D) functional terms
 between particle doses causing the maximal effect (137Cs at 160 cGy, 14Si at 4cGy and 22Ti at 6.5 cGy).
